# Dietary protein supplementation and its consequences for intake, digestion, and physical activity of a carnivorous marsupial, *Sminthopsis crassicaudata*

**DOI:** 10.1002/ece3.3843

**Published:** 2018-03-05

**Authors:** Lihong Yuan, Shawn Wilder, David Raubenheimer, Stephen J. Simpson, Michelle Shaw, Bronwyn M. McAllan

**Affiliations:** ^1^ School of Public Health Sun Yat‐Sen University Guangzhou Guangdong Province China; ^2^ School of Medical Sciences University of Sydney Sydney NSW Australia; ^3^ School of Life and Environmental Sciences and Charles Perkins Centre University of Sydney Sydney NSW Australia; ^4^ Department of Integrative Biology Oklahoma State University Stillwater OK USA; ^5^ Department of Animal Nutrition Taronga Conservation Society Mosman NSW 2088 Australia; ^6^ Present address: School of Public Health Sun Yat‐Sen University Guangzhou Guangdong Province China

**Keywords:** captive management, dietary protein, marsupial, nutritional geometry

## Abstract

Diet regulation behavior can mediate the consequences of imbalanced diets for animal well‐being, particularly for captive species that have little dietary choice. Dasyurids (carnivorous marsupials) are of conservation concern in Australia, and many species are in captive breeding programmes. However, their nutrient targets and dietary regulation behaviors are poorly understood, a limitation that may decrease the breeding success and well‐being of captive animals. We tested how dietary protein content influenced the intake and utilization of nutrients, physical activity, and body mass of fat‐tailed dunnarts *Sminthopsis crassicaudata*. Twelve adult dunnarts from six sibling pairs (one female and one male per pair) were provided ad libitum access to three diets in a repeated measures design: cat food, cat food supplemented with raw lean beef (1:1), and cat food supplemented with cooked lean beef (1:1). Food intake, activity level, and fecal output were measured daily. Dunnarts significantly decreased food intake, increased protein digestion, and physical activity, but body mass was unchanged when on the high‐protein diet compared to the normal cat food diet. These observations suggest a capacity of dunnarts to maintain constant body mass using a dynamic balance of feeding, digestion, and activity. We also found a significant effect of family, with differences between families as large as the difference between the diet treatments, suggesting a genetic component to diet selection. The nutrient regulation responses of dunnarts to high‐protein diets and the strong family effects provide important messages for the management of populations of small carnivores, including the aspects of dietary manipulation and conservation of genetic diversity.

## 1 INTRODUCTION

Dietary nutrient balance has been shown to strongly influence many life history traits of animals, including growth, reproduction, and lifespan (Koch, Ganzhorn, Rothman, Chapman, & Fichtel, 2017; López‐Alfaro, Coogan, Robbins, Fortin, & Nielsen, 2015; Simpson & Raubenheimer, 2012). There is wide variation in the availability and nutritional composition of foods in nature, and thus animals are often faced with deficient or imbalanced diets. As a consequence, many animals have evolved diet regulation behaviors to maintain a balanced nutrient intake (Knott et al., 2017; Simpson, Sibly, Lee, Behmer, & Raubenheimer, 2004). Yet, in some cases, restricted diet choices or food availability may limit the ability of animals to regulate their macronutrient intake to their desired or target levels (Moore, Wiggins, Marsh, Dearing, & Foley, 2015; Simpson et al., 2004). Dietary imbalances can have significant consequences for animal health and fitness depending on which nutrients are over‐ or underabundant. Hence, animals may respond to imbalanced diets by a combination of changes in total food intake, activity, and digestive efficiency (Irwin, Raharison, Raubenheimer, Chapman, & Rothman, 2014; Lindsay, Allen, & Major, 2015; López‐Alfaro et al., 2015; Raubenheimer, Simpson, & Tait, 2012).

Recent examination of the diets of wild animals has demonstrated that they will modify seasonal intake of available foods or modify activity to accommodate nutrient shortcomings (Coogan, Raubenheimer, Stenhouse, & Nielsen, 2014; Irwin et al., 2014; Nie et al., 2015; Rothman, Raubenheimer, & Chapman, 2011). Data are best known for primates where there is clear evidence for macronutrient balancing by precise and deliberate food choices in individuals (Felton et al., 2009; Irwin et al., 2014; Johnson, Raubenheimer, Rothman, Clarke, & Swedell, 2013; Rothman et al., 2011). However, the nutritional ecology of carnivores is much less well understood than that of herbivores, especially for vertebrate carnivores.

Until recently, it was assumed that prey quantity was in general the limiting dietary factor for predators, with quality being typically high and relatively invariant (Kohl, Coogan, & Raubenheimer, 2015). Premises concerning nutritional selection are focused around adaptations for prey capture as a primary selective force rather than for nutrient‐balancing mechanisms. This contrasts with herbivores and omnivores, which have long been considered to forage on low‐quality or variable‐quality diets, hence need nutrient‐balancing mechanisms (Kohl et al., 2015). Recent evidence, however, shows that the foods of predators are more variable than previously assumed (Tait, Raubenheimer, Stockin, Merriman, & Machovsky‐Capuska, 2014) and that this variability has fundamental fitness consequences for predators and that, such as herbivores and omnivores, they have the ability to combine nutritionally imbalanced foods in specific proportions to compose a diet that supports better performance than any of the foods alone (Kohl et al., 2015). However, very little work has been carried out examining how predators integrate different components of their nutritional biology to offset variation in dietary quality. In herbivores and omnivores, this has been termed “integrated processing response,” with many studies focussing specifically on the interaction of food selection and gut adaptations for diet balancing (Cortés, Franco, Sabat, Quijano, & Nespolo, 2011; Finotti, Moraes Santos, & Cerqueira, 2012; Naya, Bozinovic, & Karasov, 2008; Young Owl & Batzli, 1998).

Recent evidence for domesticated or captive carnivores indicates that they will self‐select specific ratios of macronutrients from nutritionally complementary foods and that these selected ratios optimize their performance (Hewson‐Hughes et al., 2011, 2013; Mayntz et al., 2009). In wild carnivores, the data concerning specific dietary selection are less forthcoming, and most data surround the species’ prey choices based on ecological interactions with other carnivores, or from investigations of human‐carnivore ecology (Bosch, Hagen‐Plantinga, & Hendriks, 2015; Newsome, Ballard, Crowther, Fleming, & Dickman, 2014; Spencer, Crowther, & Dickman, 2014). There is little analysis of nutrient content and selected preferences based on nutritional optimization in carnivores, although circumstantial evidence suggests that in the wild carnivores do feed by selecting different food items to balance their macronutrient intake (Kohl et al., 2015). In many parts of the world, both small and large carnivores are conservationally vulnerable, and knowledge of diet regulation behavior and its consequences is critical for optimal habitat conservation and captive breeding programmes alike. An example of how such knowledge into the nutritional drivers of prey selection can be relevant to practical challenges in conservation is provided by Coogan and Raubenheimer (2016), who combined in a model experimentally derived information about the macronutrients priorities of grizzly bears with data on availability of wild foods to predict the seasonal incidence of human‐bear conflict.

Carnivorous marsupials (Dasyuridae) are of particular conservation concern in Australia (Jones, Dickman, & Archer, 2003). To maintain genetic diversity and prevent species contraction, many of these carnivorous marsupials are in captive breeding programmes. In captivity, food items for dasyurids are sometimes, but not always, chosen because they are similar to food options observed to be eaten in the wild. Snapshot observations of wild foraging are extremely useful, but food prey and eating behaviors can change with seasonal prey availability and with differing seasonal physiological needs. Further, there can be substantial variation in the nutritional composition of prey items (e.g., insects Raubenheimer & Rothman, 2013; Wilder, Norris, Lee, Raubenheimer, & Simpson, 2013), including within individuals of the same prey species (Raubenheimer, Mayntz, Simpson, & Toft, 2007). Even when the types of food eaten are known, the optimal combinations of these items required by animals are often unknown. This uncertainty can lead to variation in diets fed to dasyurids within and among institutions. Providing endangered species with the optimum diets that contain essential micronutrients and proper balances of macronutrients is critical to maximize the health, increase breeding success and well‐being of animals when held in captivity (Raubenheimer et al., 2012). However, for dasyurids, little is known about their nutrient targets and diet regulation behavior, and more baseline data are needed to better understand the diet regulation behavior and requirements of carnivorous marsupials.

The fat‐tailed dunnart, *Sminthopsis crassicaudata*, is an excellent model system for examining integrated processing responses to variation in diet composition in a predator. It is a small carnivorous marsupial that is mainly distributed in arid and semiarid areas of Australia. The high variability in daily and seasonal temperature fluctuations and rainfall means that this animal is frequently faced with food shortages (Morton, 1982). Torpor, which is characterized by facultative reduction in body temperature (*T*_b_), metabolic rate, and energy expenditure, is one of the strategies that dunnarts use to survive these variable conditions (Geiser, McAllan, & Brigham, 2005). Because the fat‐tailed dunnart is locally common and we know something about their ecology and physiology, they are a good proxy for understanding physiological responses to ecological demands in other IUCN‐listed critically endangered or near threatened dunnarts (e.g., *S. aitkenii* and *S. douglasii* respectively). We know that dunnarts exposed to either reduced food availability or unpredictable presentation of food will use torpor in response to these energy bottlenecks (Munn, Kern, & McAllan, 2010). Moreover, a study on some standard diets used for two species of captive dunnarts found that when feeding on different diets, including prepared foods and insects, dunnarts consumed less food and had lower weights on diets that had relatively higher protein content (Stannard, McAllan, & Old, 2014) However, this study only investigated the individual choice of common captive diets rather than specific manipulations of macronutrients (Stannard et al., 2014). Recently, we demonstrated that the fat‐tailed dunnart will select for more fat in the diet if given a choice, but that energy balance was maintained by increasing their activity patterns (Wilder et al., 2016). We observed that selection for protein did differ if the source of the protein was different (Wilder et al., 2016). As diets for captive carnivores are usually low in fat and high in protein, the source of protein deserves further examination.

In this study, we tested the consequences of manipulating dietary protein content for the total food intake, activity level, and fecal production of captive dunnarts. Because some captive programs cook protein for bacterial control to improve health and welfare for captive animals, we also tested how cooking protein affected intake, activity, and defecation of fat‐tailed dunnarts. Cooking significantly affects myofibrillar protein susceptibility to proteases and can decrease passage time of proteins in the gut (Bax et al., 2013; Santé‐Lhoutellier, Astruc, Marinova, Greve, & Gatellier, 2008). Protein content was manipulated using extra lean ground beef. The dunnarts were fed three diets over the course of alternating feedings: standard cat food diet, 1:1 mixture of cat food and raw beef, and 1:1 mixture of cat food and cooked beef. Our study used six pairs of male and female siblings in a repeated measures design, which also allowed us to test the separate effects of sex and family on response variables and if these factors interacted with an individual's response to the diet. Our aims were to determine whether dunnarts preferred high‐protein diets over the control diets and whether the extra protein in the diet affected behavioral outcomes. These outcomes have important implications for the management of captive carnivores. Not only does this extend the integrated processing response paradigm to predators, but it also builds on that paradigm through applying it to a species with discrete nutrient selection, energy storage, energy conservation, and energy expenditure mechanisms.

## 2 MATERIALS AND METHODS

### 2.1 Animal housing

Twelve adult fat‐tailed dunnarts (*Sminthopsis crassicaudata*), aged from 15 to 17 months and with body mass 14.29 ± 1.55 g and tail width 5.61 ± 0.74 mm, captive‐bred at the University of Sydney were used for this study. These animals consisted of six pairs of siblings (one female and one male of each sibling pair). The use of siblings allowed us to control for family effects in statistical analyses. Throughout the study, dunnarts were housed individually in cages (internal dimension 20 cm × 20 cm × 30 cm) with nest boxes (two clean cardboard rolls which were sealed at one end), and under natural photoperiod with ambient temperature (*T*_a_) at 20 ± 2°C. Animals were fed each day and had access to water ad libitum. The eye surface temperature was measured in the morning (8:00–8:30 a.m.) of every second day by placing an infrared digital thermometer (SE‐100, SEIN ELECTRONICS) about 2 cm above the eye with the temperature measured nearest to 0.1°C (Song & Geiser, 1997), and all animals were normothermic throughout the whole study. The temperature of the eye has been demonstrated to be a good proxy for body temperature in a closely related species *Sminthopsis macroura* (Song & Geiser, 1997). The study was approved by the Animal Ethics Committee of University of Sydney (K25/5‐2013/3/6000).

### 2.2 Diets

To determine the effect of dietary protein content on food intake and activity, dunnarts were offered two major diets: control diet of low‐protein food (Commercial cat food, “Whiskas,” jellymeat variety with 7% protein and 5.5% fat by wet mass) and high‐protein diet: a 1:1 meat‐supplemented cat food (50% extra lean (“3% fat”) beef mince: the content of 21% protein and 3% fat on a wet mass basis; 50% cat food). Beef mince was frozen at −20°C for >1 month as a precaution against pathogen exposure. However, to determine the effect of cooking on the food intake and digestion of high‐protein diet, the animals were split into two groups (three pairs of siblings each) and fed with cooked high‐protein diet (cooked in microwave for 50 s, the beef mince was browned, but not dessicated) and uncooked high‐protein diet (raw mixture), respectively. Thus, a total of three diets, an uncooked high‐protein diet, a cooked high‐protein diet, and one control diet, were used across the course of the experiment. Care was taken to include all meat components in both treatments, for example not to discard melted fat from cooked meat thus ensuring that the foods differed only in the thermal treatment of identical ingredients. Furthermore, the cooked and raw high‐protein diets were switched between two groups after a washout period (control diet) to remove the effects of presenting the food in a particular order. Diets were blended in a domestic blender to thoroughly mix the foods.

### 2.3 Feeding regime

**Figure 1:**
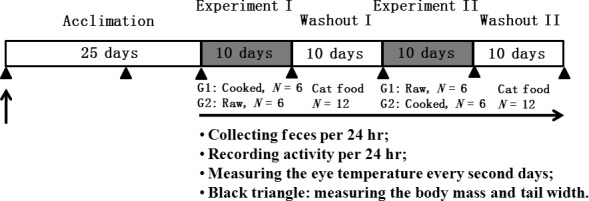
Illustration of experimental design

The animals were fed low‐protein diet (cat food, *N* = 12) 25 days for acclimation, followed by the 10 days of experimental period I in which animals were fed high‐protein diet (cooked diet, *N* = 6; raw diet, *N* = 6), 10 days of washout period I fed with cat food (*N* = 12), 10 days of experimental period II of high‐protein diet (switch of cooked and raw diets between two groups, *N* = 6 of each diet), and another 10 d of washout period II (cat food, *N* = 12). A crossover design was used where the animals that were exposed to the cooked high‐protein diet in the first experimental period were exposed to the uncooked high‐protein diet in the second experimental period. Siblings of these animals were exposed to the diets in the reverse order during the experimental periods. During periods of control diet, two control dishes were used to calculate the water loss rate from food dishes each day, and during high‐protein diets, four control dishes—two of cooked and two of raw—were used as water loss controls each day. Throughout the study, animals had access to food and drinking water ad libitum, and as per normal captive colony, husbandry was provided with vitamins and mineral powder mixed in the food on Wednesdays and calcium on Sundays. The animals were fed each day to excess, from previous studies, we know the maximum amount of food dunnarts will eat each day (Munn et al., 2010; Stannard et al., 2014), and all uneaten food was collected each day for later analysis. The detailed experimental design is given in Figure 1. The following equation was used to calculate the daily food intake:

Dehydration=(a−b)/(a−c)

Food intake=a−b−d∗(b−c)

*a* = Initial weight of food with dish; *b* = Remaining weight of food with dish; *c* = Average weight of empty dish (0.22 g); *d* = Dehydration.

### 2.4 Feces collection and assay

Feces were collected daily before feeding. Protein content of the fecal bolus was measured on a subsample of feces for all animals on days 5–8 of the first and second experimental periods (Experiments I and II), and the washout period I. Briefly, approximately 15–25 mg of dry feces was digested in 2 ml of 0.1 mol/L NaOH for 30 min at 80°C. The protein in the samples was measured using a modification of the Bradford method (Barry & Wilder, 2013). Briefly, purified by centrifuging at 18000 g for 10 min, removing the supernatant, precipitating protein with 100% trichloroacetic acid (TCA), centrifuging to compact the protein into a pellet, and washing with −20°C acetone to remove any residual TCA. Protein pellets were re‐suspended in 2 ml of 0.1 mol/L NaOH and assayed in a Direct Detect Spectrophotometer (Merck KGaA, Germany) to quantify protein concentrations.

### 2.5 Body mass and tail width measurements

The body mass and tail width of dunnarts were measured at the beginning, the middle, and the end of acclimation, and at the end of each experimental or washout period using an electronic balance to 0.01 g (Industrial and Scientific Supply Company Pty Ltd). Tail widths, which are an indicator of fattening and body condition (McAllan, Feay, Bradley, & Geiser, 2012), were measured using vernier calipers (Dick Smith Electronic calipers).

### 2.6 Activity measurement

For each animal, a 12‐cm diameter wheel with pedometer (CATEYE CC‐VL820) was provided to record the daily activity. Distance run (km/day), time spent running (seconds/day), average and maximum speed (km/hr) were measured each day.

### 2.7 Data analysis

Response variables (intake, traveled distance, fecal dry mass, fecal percent protein, fecal protein mass, and change in body mass) were analyzed using repeated measures analysis of variance (RMANOVA) using SPSS. Separate analyses were conducted for comparisons of the raw versus cooked meat supplementation, and for cat food versus cat food supplemented with ground beef (both raw and cooked combined). For the raw versus cooked meat supplementation comparisons, the main between‐subjects effects in the model were as follows: treatment, sex, treatment*sex, family, and period. Hereafter, “Treatment” was referred as diets (e.g., raw vs. cooked meat supplementation, or cat food vs. cat food supplemented with ground beef); “Period” was a block of 10 days that the dunnarts were on one of the diets; “Time” indicated the comparing measurements on different days in the same period. For the cat food versus cat food supplemented with meat comparisons, the main between‐subjects effects in the model were as follows: treatment, sex, treatment*sex, and family. For the cat food versus cat food supplemented with meat comparisons, we presented results comparing experiment I (both raw and cooked combined) with the washout period I. Qualitatively similar results were obtained when comparing experiment II with washout period II, and when comparing both meat supplementations with both washout periods; although for the latter comparison, the degrees of freedom were artificially high. Univariate analysis was carried out, and all data were presented as mean ± *SE*. Pearson's correlation analysis was conducted to deduce the relationship among dependent variables.

## 3 RESULTS

### 3.1 Food and nutrient intake and utilization

**Figure 2:**
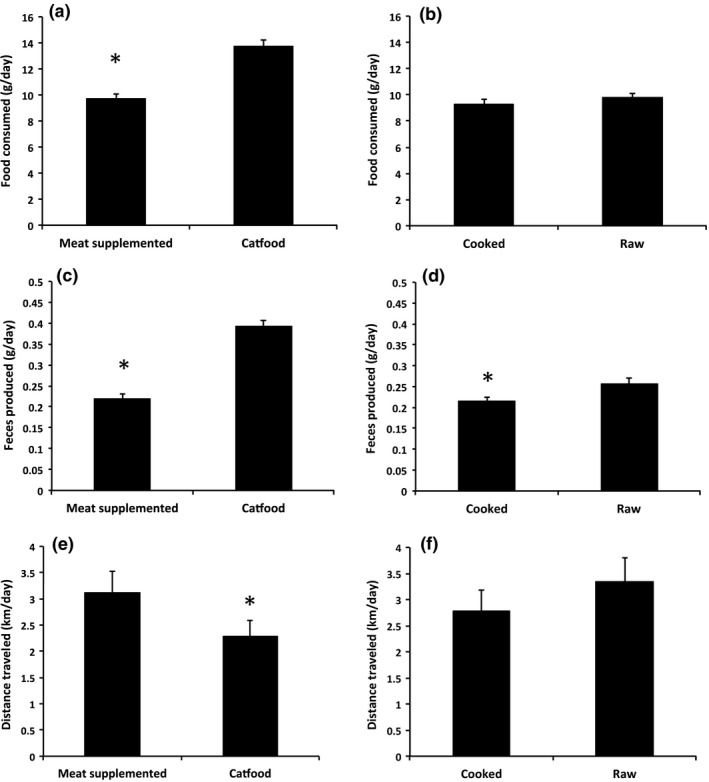
Food consumption, fecal output, and distance traveled when animals were exposed to different diets. Panels (a) and (b) are food consumption (g); panels (c) and (d) are fecal output (g); and panels (e) and (f) are distance run per day (km). Panels A, (c), and (e) compare high‐protein versus cat food diets, and panels (b), (d), and (f) compare the cooked versus uncooked high‐protein diets. Data are means ± SEM, asterisks indicate data are significantly different

**Figure 3:**
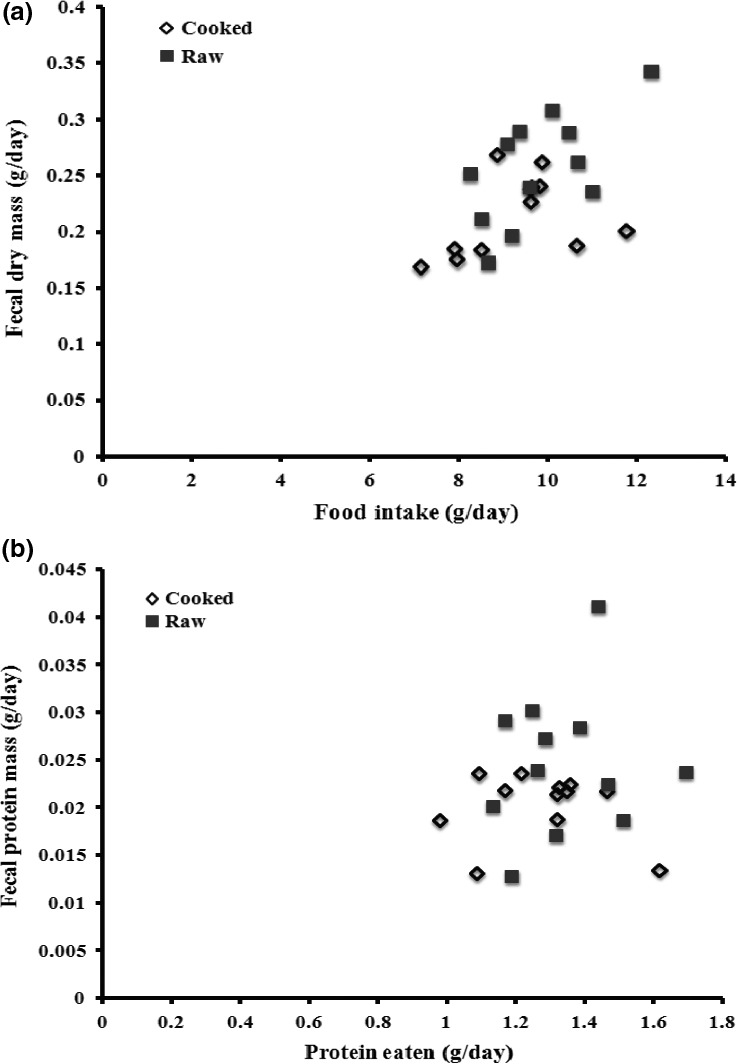
General utilization plot for food intake and protein utilization of cooked versus raw meat‐supplemented cat food. (a) Food intake and fecal dry mass; (b) Protein utilization

There was no significant main effect of raw versus cooked meat on the dunnarts’ food intake, and food intake was similar for eight of the ten feedings (Table 1; Figure 2b). There was, however, a significant treatment by time interaction in which there were two time periods (days 2 and 4) where dunnarts ate more of the raw than the cooked diets (Table 1, *p* < .05). There was also a significant treatment by time interaction in fecal dry mass production with higher fecal production by dunnarts fed raw diets for most, but not all, days (Table 2). There were no major patterns when comparing either total food or protein utilization plots, which examine the relationships between the amount eaten and fecal content (Table 1; Figure 3 and see Raubenheimer & Simpson, 1994). There was a significant main effect of sex (i.e., independent of diet) on intake (i.e., males ate more than females) and a significant main effects of family on intake and fecal dry mass (Tables 1 and 2). Food intake of some families was up to 50% higher than that of other families.

**Table 1:** Summary of statistical analyses for food intake (g/day), distance traveled (km/day), and body mass (g)

	Intake			Distance			Change in body mass		
	*df*	*F*	*p*	*df*	*F*	*p*	*df*	*F*	*p*
Raw versus cooked diets									
Between‐subjects									
Treatment	1, 14	0.26	.07	1,13	0.14	.2	1	0.12	.74
Sex	1, 14	0.84	.004^b^	1,13	0.62	.01^b^	1	0.98	.34
Treatment × sex	1, 14	0.12	.21	1,13	0.003	.84	1	3.94	.07
Family	5, 14	4.2	.0001^b^	5,13	3.36	.0008^b^	5	1.33	.31
Period	1, 14	0.19	.13	1,13	5.00E−04	.94	1	0.74	.41
Within‐subjects									
Time	9, 6	1.88	.41	9,5	4.44	.17	—	—	—
Time × treatment	9, 6	13.01	.008^b^	9,5	1.38	.66	—	—	—
Time × sex	9, 6	3.5	.16	9,5	1.56	.6	—	—	—
Time × treatment × sex	9, 6	3.04	.2	9,5	1.53	.61	—	—	—
Time × family	45, 30	1.61	.09	45,25	0.95	.57	—	—	—
Time × period	9, 6	1.72	.45	9,5	1.38	.66	—	—	—
High‐protein diet versus cat food (Experiment I vs. Washout I)									
Between‐subjects									
Treatment	1, 15	13.39	<.0001^b^	1,14	0.4	.03^a^	1	2.93	.11
Sex	1, 15	0.21	.09	1,14	0.81	.005^b^	1	0.73	.41
Treatment × sex	1, 15	0.11	.22	1,14	0.28	.07	1	0.38	.55
Family	5, 15	4.31	<.0001^b^	5,14	2.66	.001^b^	5	0.43	.82
Within‐subjects									
Time	9, 7	4.86	.05^a^	9,6	10. 4	.01^b^	—	—	—
Time × treatment	9, 7	3.76	.08	9,6	19.69	.003^b^	—	—	—
Time × sex	9, 7	6.86	.02^a^	9,6	4.13	.12	—	—	—
Time × treatment × sex	9, 7	2.48	.2	9,6	1.54	.51	—	—	—
Time × family	45, 34	1.46	.12	45,30	2.18	.01^b^	—	—	—

Cat food: commercial Whiskas’ jellymeat; High‐protein diets: 1:1 meat‐supplemented cat food (both raw and cooked combined); Raw/Cooked diets: raw/cooked high‐protein diets. Sex: male and female; Family: six pairs of siblings from six pairs of different parents. Treatment: diets of raw versus cooked meat supplementation, or cat food versus cat food supplemented with ground beef; Period: a block of 10 days that the dunnarts were on one of the diets; Time: comparing the measurements on different days in the same period.

a *p *<* *.05.

b *p *<* *.01.

**Table 2:** Summary of statistical analyses for data on fecal analyses

	Fecal dry mass			Fecal percent protein			Fecal protein mass		
	*df*	*F*	*p*	*df*	*F*	*p*	*df*	*F*	*p*
Raw versus cooked diets									
Between‐subjects									
Treatment	1,14	1.07	.002^a^	1,14	0.003	.84	1,14	0.31	.06
Sex	1,14	2.00E−04	.96	1,14	0.01	.71	1,14	0.05	.4
Treatment × sex	1,14	0.04	.48	1,14	0.02	.65	1,14	0.07	.35
Family	5,14	2.15	.004^a^	5,14	0.45	.33	5,14	0.5	.28
Period	1,14	0.16	.15	1,14	0.24	.09	1,14	0.39	.04*
Within‐subjects									
Time	7,8	17.17	.0002^a^	3,12	1.18	.02*	3,12	0.42	.23
Time × treatment	7,8	3.25	.04*	3,12	0.07	.85	3,12	0.11	.72
Time × sex	7,8	1.81	.16	3,12	0.17	.59	3,12	0.04	.93
Time × treatment × sex	7,8	1.35	.28	3,12	0.26	.41	3,12	0.31	.34
Time × family	35,36	1.32	.2	15,33	1.98	.049*	15,33	2.04	.04*
Time × period	7,8	50.11	<.0001^a^	3,12	5.28	<.0001^a^	3,12	1.03	.03
High‐protein diet versus cat food (Experiment I vs. washout I)									
Between‐subjects									
Treatment	1,14	13.62	<.0001^a^	1,13	1.32	.001^a^	1,13	9.93	<.0001^a^
Sex	1,14	0.17	.15	1,13	0.46	.03*	1,13	0.58	.02*
Treatment × sex	1,14	0.18	.13	1,13	0.21	.12	1,13	0.67	.01^a^
Family	5,14	2.31	.003^a^	5,13	0.32	.54	5,13	0.74	.16
Within‐subjects									
Time	7,8	25.29	<.0001^a^	3,11	0.61	.14	3,11	0.27	.43
Time × treatment	7,8	11.32	.0009^a^	3,11	2.08	.005^a^	3,11	0.43	.25
Time × sex	7,8	0.67	.63	3,11	0.3	.39	3,11	0.16	.63
Time × treatment × sex	7,8	0.4	.84	3,11	0.31	.37	3,11	0.35	.33
Time × family	35,36	0.92	.6	15,31	1.35	.24	15,30	0.83	.64

Cat food: commercial Whiskas’ jellymeat; High‐protein diets: 1:1 meat‐supplemented cat food (both raw and cooked combined); Raw/Cooked diets: raw/cooked high‐protein diets. Sex: male and female; Family: six pairs of siblings from six pairs of different parents. Treatment: diets of raw versus cooked meat supplementation, or cat food versus cat food supplemented with ground beef; Period: a block of 10 days that the dunnarts were on one of the diets; Time: comparing the measurements on different days in the same period.

*p *<* *.05.

a *p *<* *.01.

**Figure 4:**
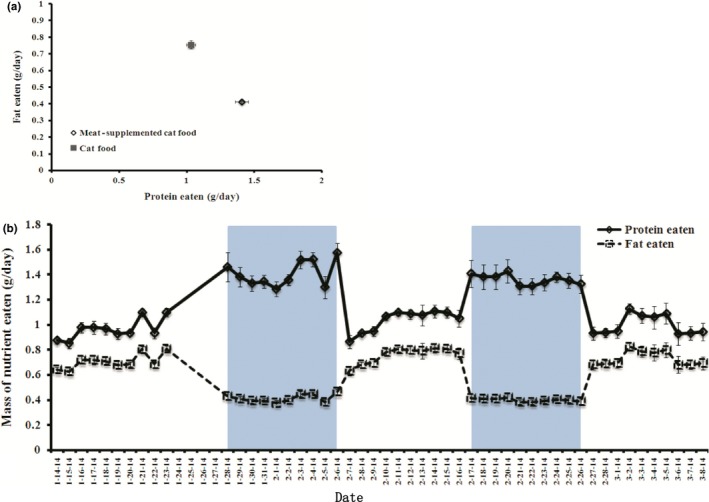
Plots of protein and fat eaten of cat food versus high‐protein diets (1:1 meat‐supplemented cat food, raw and cooked combined). (a) General plot of protein and fat eaten. (b) A time series of protein and fat consumption from the last 10 days of the initial acclimation through the final washout period. The periods of high‐protein diets are shown by the gray background

**Figure 5:**
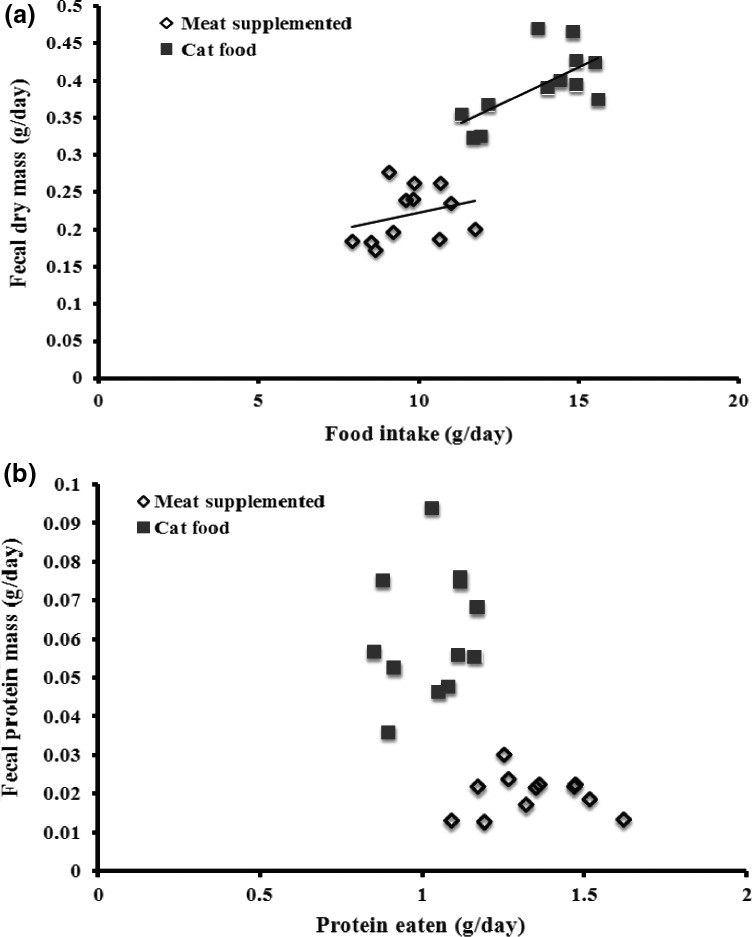
General utilization plot for food intake and protein utilization of cat food versus high‐protein diets (1:1 meat‐supplemented cat food, raw and cooked combined). (a) Food intake and fecal dry mass; (b) Protein utilization per gram fecal dry mass

When the cat food diet was compared to the high‐protein diets (raw and cooked combined), dunnarts on the high‐protein diet ate significantly less total food, ran a further distance, produced less feces, which had lower protein concentration than when on the cat food diet (Table 2; Figure 2). Analysis of nutrient intake showed that dunnarts on the protein‐supplemented diet ingested more protein and less fat than dunnarts on the cat food diet (Figure 4). In utilization plots examining overall intake, dunnarts on the cat food diet ate more total food and produced a higher total fecal mass than dunnarts on the protein‐supplemented diet (Tables 1 and 2; Figure 5a). In protein utilization plots, dunnarts on the cat food diet ate less total protein but produced more protein in feces than dunnarts on the protein‐supplemented diet (Figure 5b). There were significant main effects of sex on fecal protein content (males < females for both measures) and significant effects of family on intake and fecal dry mass.

### 3.2 Body mass changes with diets

Body mass (g) showed a significantly positive correlation with tail width (mm, *r* = .44, *p* < .01), thus we chose body mass for further analysis. When comparing raw versus cooked diet, treatment (diets), sex, and family had no significant effects on the change in body mass (Table 1). Similarly, treatment (diets), sex, and family had no significant effects on the change in body mass comparing cat food versus high‐protein diets (Table 1).

### 3.3 Physical activity

Running distance (mean 2.64 ± 1.48 km/day) correlated positively with Log10‐transformed running time (mean 9.07 ± 0.61 sec/d; *r* = .82, *p* < .01), average speed (0.91 ± 0.16 km/hr; *r* = .81, *p* < .01; Table 3), and maximum speed (mean2.66 ± 0.30 km/hr; *r* = .30, *p* < .01; Table 3). To avoid redundancy, we therefore used only the daily distance in the analysis of dunnarts’ physical activity.

**Table 3:** Summary of the regression analysis between food intake and activity of three diets

Activity	Cat food (washout I)			Cooked diet			Raw diet		
	*df*	*F*	*p*	*df*	*F*	*p*	*df*	*F*	*p*
Sex	1	0.46	.52	1	1.25	.3	1	0.77	.41
Intake	1	6.21	.04^a^	1	7.58	.02^a^	1	9.01	.02^a^
Sex^a^Intake	1	0.14	.72	1	0.38	.55	1	2.05	.19

a *p *<* *.05.

When comparing raw and cooked meat diets, there was no effect of diet either as a main effect or in interaction with other factors on total distance traveled (Table 1; Figure 2f). However, sex and family (main effects independent of diet) significantly affected activity levels, and males ran significantly further than females on both cooked (males: 3.32 ± 0.2 km/day vs. females: 2.25 ± 0.2 km/day, *p* < .01) and raw diets (males: 3.72 ± 0.21 km/day vs. females: 2.94 ± 0.21 km/day, *p* = .01). The effect of family on distance traveled was large, with some families traveling over four times greater distance per night than other families (e.g., <1 vs. >4 km/day).

When comparing the effects of cat food with high‐protein diets (raw and cooked combined), the effects of diet (treatment), sex, family, and time on activity were significant (Table 1). The activity of animals on high‐protein diets was significantly higher than that of animals on cat food (3.01 ± 0.1 km/day vs. 2.34 ± 0.1 km/day, *p* < .01, Figure 2e), and also males’ activity was significantly higher than that of females on both high‐protein (males: 3.52 ± 0.15 km/day vs. females: 2.6 ± 0.15 km/day, *p* < .01) and cat food diets (males: 2.58 ± 0.12 km/day vs. females: 2.1 ± 0.12 km/day, *p* < .01). Again, the effect of family was large, with some families traveling 2–3 times further per night than other families.

## 4 DISCUSSION

Our results show that dietary protein supplementation had significant effects on the food intake, fecal production, and activity level of fat‐tailed dunnarts. Dunnarts feeding on the protein‐supplemented food ingested less overall food, produced less feces, and were significantly more active. Whether or not the protein supplement, extra lean ground beef was cooked had very little effect on dunnart intake, fecal production, or activity level. These results suggest that dietary macronutrient balance may be important for marsupial carnivores, as has been observed in a wide range of eutherians and in many invertebrates (Simpson & Raubenheimer, 2012). In addition, these results demonstrate that dunnarts adjust their intake and activity level depending on their diet. Rather than gaining weight when fed the food with higher total nutrient content, which also had higher total energy content, dunnarts decreased their consumption of the diet and increased their activity level, resulting in no significant changes in weight when on the different diets. In addition to the nutritional benefits of higher nutrient food, increased activity on the high‐protein diet could contribute to dunnart health in captivity by helping animals maintain muscle mass and healthy metabolism and might also provide a significant form of enrichment. Further data are needed on the nutrient content of potential prey and how prey nutrients vary spatially and temporally in nature to determine the ecological consequences of these effects.

We found that dunnarts were normothermic throughout the experiment (data not shown), indicating that they had enough energy available at all times for adequate metabolism and so did not use torpor. However, dunnarts ate less of the high‐protein diet (raw and cooked combined, Figure 5a) when compared with intake of the low‐protein diet (normal cat food). Lower food intake is probably due to the higher nutrient density of high‐protein diets, and also the much higher protein:lipid content (14.5%:4.25%) than regular cat food (7%:5.5%). Our analysis of nutrient intakes showed that dunnarts ate more protein and less lipid on the protein‐supplemented diet relative to the cat food diet (Figure 4). By eating more protein and less lipids during exposure to high‐protein diets, dunnarts may be satisfying their overall energy requirements by balancing between overconsumption of protein and underconsumption of lipid, as has been observed in some eutherian carnivores (Hewson‐Hughes et al., 2011; Mayntz et al., 2009).

Other studies have shown that adding dietary lipids can affect both energetics and torpor use in eutherians and marsupials alike (Bozinovic & Méndez, 1997; Contreras, Franco, Place, & Nespolo, 2014; Faherty, Campbell, Hilbig, & Yoder, 2017; Geiser, Klingenspor, & McAllan, 2013; Geiser, McAllan, Kenagy, & Hiebert, 2007; Geiser, Stahl, & Learmonth, 1992). However, polyunsaturation of lipids in the diet can strongly influence torpor use and torpor duration in mammals whose omnivorous or granivorous diets regularly include natural foods rich in these nutrients (Bozinovic & Méndez, 1997; Geiser et al., 1992, 2007, 2013). Few lipid‐supplementation studies have been performed on strict carnivores to determine the effects of diet on torpor use in these mammals (Wilder et al., 2016). Our previous study on dunnarts found that they chose to eat diets with fat; however, body mass was not affected and activity was moderated according to the diet (Wilder et al., 2016). Similarly, in the present study, metabolism and activity were adjusted when presented with a high‐protein diet. The data would suggest that providing basic macronutrients are available, and providing activity can be maintained, animals will self‐select both food eaten and activity outputs. These are important considerations for the promotion of optimum body condition in captivity and in the wild.

We tested both raw and cooked meat supplements because we predicted that cooking the supplement would increase the digestibility of the protein. While dunnarts on the raw diet produced more feces, we found no significant difference in the percent protein or mass of protein in the dunnart feces between raw and cooked supplemented diets. However, we detected apparent differences in digestibility when comparing the cat food and cat food supplemented with meat diets. Dunnarts produced a lower mass of feces and feces with lower percent protein and mass of protein on the high‐protein diet, despite consuming more protein on this diet relative to the cat food diet. One potential explanation for the higher apparent digestibility of protein when on the protein‐supplemented diet is that the protein in the meat added to the food was more easily digested by dunnarts than the protein in cat food. Another possible explanation is that dunnarts increased their digestion of protein on the protein‐supplemented diet to compensate for the lack of energy from lipid on this diet. While it is likely a combination of these two factors, more detailed studies of the digestibility of the proteins present in the cat food and meat as well as the capacity of dunnarts to alter their protein digestion efficiency are needed to test their relative importance.

We also found that the animals’ activity on high‐protein diets was significantly higher than when they were given cat food alone, whereas no significant difference was observed between the raw diet and cooked diet (Table 1). Possible explanations for the higher activity level on the meat‐supplemented diet could be that the animals had more time to run as they ate less food, and/or the higher activity level was a searching strategy to find a food with a better nutrient balance (e.g., less protein and more lipid or carbohydrates). Increased locomotion associated with nutritional imbalance has been observed in other animals, including large‐scale migrations in the wild (Simpson, Sword, Lorch, & Couzin, 2006). Studies using a nutritional geometry approach to quantify the intake target of animals would be useful for examining the self‐selected nutrient intake of dunnarts and how that compares to the diets used in this study (Raubenheimer, 2011). If the self‐selected diet were closer to the protein‐supplemented food, it would provide support for dunnarts being nutritionally satiated and thus allocating more effort to other activities like running. Conversely, if the self‐selected diet was closer to the cat food, then dunnarts may have been unable to balance for nutritional requirements using the food and thus continue searching for alternate foods. Maintenance of a constant weight with little or no torpor and high activity levels suggests that the dunnarts may have been satisfied with the protein‐supplemented diet. It may also be important to test the ecological consequences of the effect of diet on locomotor behavior as higher activity levels could result in greater exposure to predators.

Another important observation is the highly significant family effects and the large size of these effects. We found that the difference between two families was sometimes larger than the average difference between two dietary treatments. These large and significant family effects are somewhat surprising, but also consistent with the profound genetic influence on metabolic allocation strategies (Madon‐Simon et al., 2014). We believe they indicate three important messages for the study and management of captive populations of small carnivores. First, observations on small groups (e.g., pairs) of captive carnivores may not be representative of the larger population, and thus planning for dietary manipulation for breeding or activity outcomes may not be translated from one small group to another small group. Second, it suggests that genetic diversity in nutritional responses which may prove important if captive populations are being prepared for release back into the wild. Researchers need to consider the nutritional impact of releasing differing phenotypes into energy rich or poor situations as dispersal, mate seeking, and long‐term survival may all be affected. Finally, family background is an important factor that needs to be explicitly included in future studies of fat‐tailed dunnarts. Otherwise, variation due to family background could mask potentially significant treatment effects. Further study of the Nutritional Ecology of marsupial carnivo res may be valuable for improving diets fed to these animals in captive breeding programs, especially endangered marsupial carnivores (e.g., Tasmanian devils, several species of quolls).

Our study demonstrated that dunnarts have a capacity to maintain constant body mass using a dynamic balance of feeding, digestion, and activity. We also found a significant effect of family, with differences between families as large as the difference between the diet treatments. The nutrient regulation responses of dunnarts to high‐protein diets and the strong family effects provide important messages for the management of captive populations of small carnivores, including the aspects of dietary manipulation and conservation of genetic diversity. Our results also have implications for understanding fitness and population dynamics of these carnivores in nature as factors that cause changes in the nutrient content of prey (e.g., invasive species, climate change) could result in a cascade of physiological and behavioral changes in individuals.

## AVAILABILITY OF DATA AND MATERIALS

All data generated or analyzed during this study are included in this published article.

## CONFLICT OF INTEREST

The authors declare that they have no conflict of interests.

## AUTHOR CONTRIBUTIONS

SW, DR, SJS, MS, BMM, and LHY all provided input into the planning and execution of the experiments and all authors contributed to the manuscript preparation.
